# OligoDOM^TM^: a T-cell response-enhancing platform applied to cancer immunotherapy

**DOI:** 10.3389/fimmu.2025.1549112

**Published:** 2025-03-14

**Authors:** Judith Del Campo, Séverine Valsesia, Elsa Nikly, Roberto Ruiu, Antonella Iacoviello, Elena Quaglino, Federica Cavallo, Dalil Hannani, Emilie Boucher, Florence Nicolas, Alexandre Le Vert, Francesco Doro

**Affiliations:** ^1^ Osivax, Lyon, France; ^2^ Department of Molecular Biotechnology and Health Sciences, Molecular Biotechnology Center “Guido Tarone”, University of Turin, Turin, Italy; ^3^ CNRS, UMR 5525, VetAgro Sup, Grenoble INP, TIMC, University Grenoble Alpes, Grenoble, France

**Keywords:** immunotherapy, cancer, t cells, mRNA, nanoparticles

## Abstract

**Background:**

Neoepitopes derived (0) from tumors are attractive cancer immunotherapy targets, especially when combined with immune checkpoint inhibitors (CPIs). Vaccines using lipid nanoparticle (LNP)-encapsulated mRNA to deliver neoepitopes have shown encouraging results in patients and animal models, due to T cell-dependent responses. However, a low mutational burden is often a predictor of poor CPI response: the immune response against the few available mutations can be insufficient. An enhanced response to these few mutations could increase CPI efficacy. Here, we investigate the potential of oligoDOM™, a self-assembling sequence, to improve neoepitope immunogenicity and antitumor efficacy in murine cancer models.

**Methods:**

LNP-formulated mRNA constructs encoding short epitope strings fused with oligoDOM™ were tested. Immune responses in mice were compared between constructs with oligoDOM™ and their controls. Specific T-cell responses against four tumor models (MC38, CT26, TC-1, B16-OVA) were measured using ELISpot in naïve mice. Two models (TC-1 and B16-OVA) were further selected for tumor growth efficacy testing.

**Results:**

LNP-formulated neoepitope-oligoDOM™ mRNA constructs induced a significantly superior immune response as compared with the control groups in four neoantigens tested. This increased specific immunogenicity is linked to antitumor growth effects in murine syngeneic cancer models such as the B16-OVA and TC-1. The induced T-cell immune response significantly correlated with tumor growth rate reduction.

**Discussion:**

Combining oligoDOM™ and LNP-mRNA technologies offers a versatile platform that allows for efficient short neoepitope strings delivery. This approach represents a feasible, potentially effective strategy for personalized cancer immunotherapy.

## Introduction

Cancer is a leading cause of death worldwide, identified by WHO as the first or second leading cause of death before the age of 70 in 112 of 183 countries between 2000 and 2019 (1). Despite advanced chemotherapy and radiotherapy, some cancers, such as lung or pancreatic cancers, still have low 5-year survival rates (2).

Novel immunotherapies, including immune checkpoint inhibitors (CPIs), prevent cancer cells from evading the immune system (3, 4). The use of monoclonal antibodies targeting the CTLA-4 and PD-1 pathways has been approved for various cancer types (5). Combining neoepitope-based vaccines with CPIs is paving the way for personalized immunotherapies, enhancing tumor-specific T-cell generation and effector functions. Neoepitope vaccination may be tailored for each patient and has proven its ability to significantly increase the survival rates when combined with CPI treatments (6–8).

mRNA is a key platform for delivering neoepitopes, offering the versatility needed for personalized therapies. However, preclinical studies have shown that a strong immune response against specific mutations does not always translate into effective tumor growth inhibition. Long neoepitope strings are indeed required to broaden the immune response and observe efficacy (9, 10), which is challenging for tumors with a low mutational burden, where the scarcity of mutations can impair the overall efficacy of the combination treatments with CPIs and neoepitope vaccination (11).

OSIVAX’s oligoDOM™ (OVX313) is a platform based on the heptamerization domain of the chicken C4b-binding protein (C4BP), which can self-assemble to provide multiple antigen presentations for an enhanced immune response (12, 13). We previously demonstrated that oligoDOM™ can provide broad T-cell activation against influenza nucleoprotein (NP) in clinical trials with a strong safety profile in over a thousand subjects (14–16). Currently, a severe acute respiratory syndrome coronavirus 2 (SarsCov2) and a human papillomavirus (HPV) program incorporating oligoDOM™ are in phase I clinical trials (CT numbers: 2023-506396-94-00 and 2021-002584-22), following promising preclinical results (17, 18).

Previous evidence of an enhanced CD8 T-cell activation against viral antigens triggered by oligoDOM™ inspired the idea of combining this platform with tumor neoepitopes to extend its applicability to cancer treatment. The oligoDOM™ sequence was encoded into an mRNA construct and fused with one or two neoepitopes in series to test for superior efficacy against tumor growth compared with neoepitopes delivered alone. Here, we demonstrate how oligoDOM™ significantly improved the T-cell immune response against four different mouse cancer-associated epitope combinations and enhanced the efficacy of short neoepitope strings in two murine tumor models, both in therapeutic (in a B16-OVA model) and prophylactic (in an HPV-TC-1 model) settings.

## Methods

### mRNA constructs

**Figure 1:**
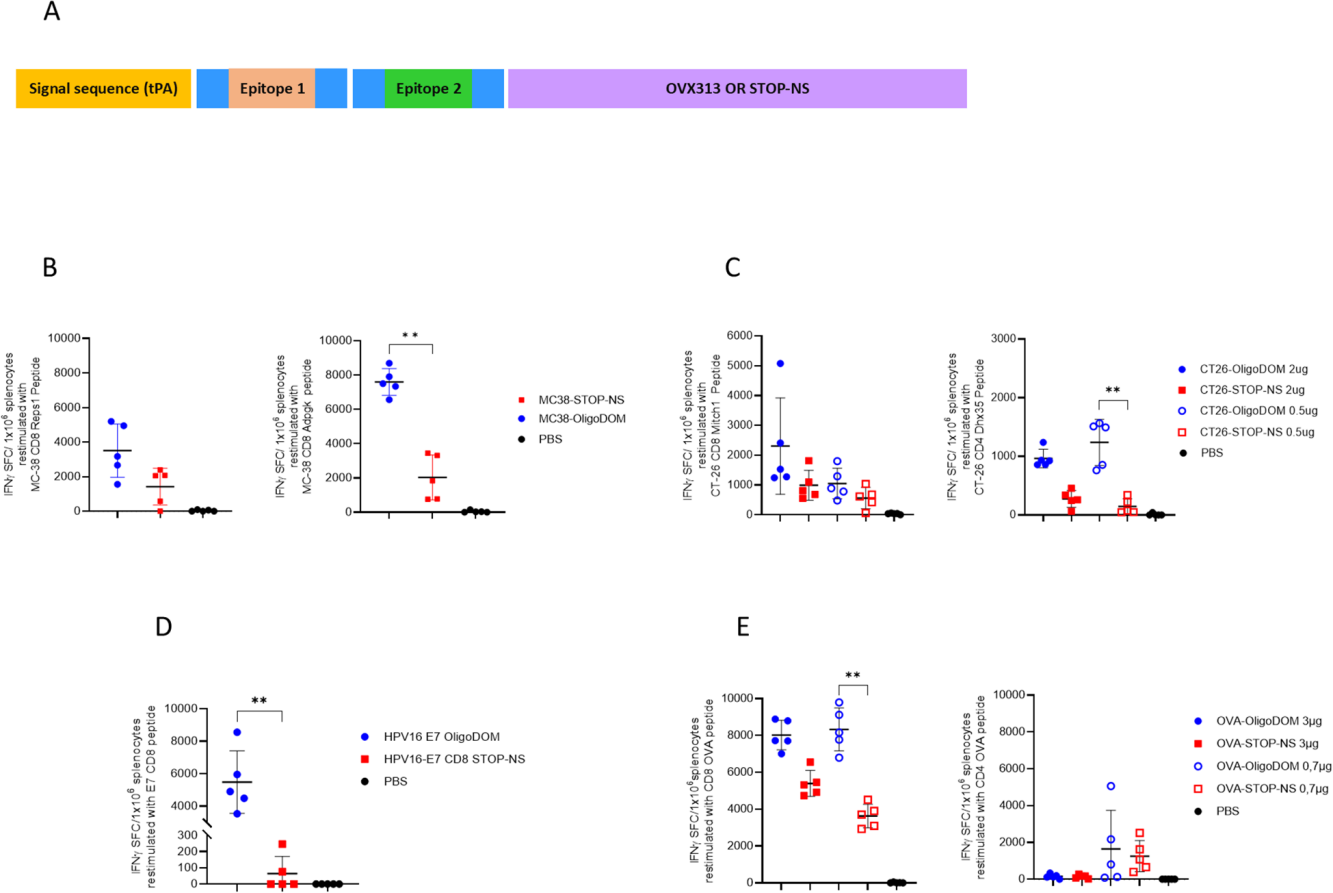
OligoDOM™ in mRNA constructs induces stronger specific T cells IFNg response in the spleen compared to the constructs without this sequence. Naïve C57BL/6 mice (n = 5) were immunized twice (D0, D21) with 50 µL of different mRNA constructs by the IM route. **(A)** Schematic representation of the constructs used for this study. **(B–E)** Immune responses evaluated 7 days after the second immunization by measuring specific IFNγ secreting splenocytes (spot-forming cells (SFCs)/1 × 10^6^ cells) using ELISpot after restimulation with specific peptides for 24 hours: **(B)** MC38 CD8-epitopes Reps1 or Adpgk, **(C)** CT26 epitopes Mitch 1 (CD8) or Dhx35 (CD4), **(D)** E7 CD8-epitope and **(E)** OVA CD8- or CD4-epitopes. Individual data, mean (line), and SD are represented. Differences were assessed by one-way ANOVA followed by Dunn’s multiple comparison test. **p < 0.01.

Constructs containing epitopes from four tumor models were created (**Table 1**, **Figure 1A**). Each construct comprised one or two epitopes in series, with each epitope flanked by five more amino acids at both N- and C-termini, corresponding to the flanking regions present in the full-length protein the epitopes come from. The exception was the CT26 epitopes, which were 20-amino acids long as reported in the literature (10). A tissue plasminogen activator (tPA) secretion sequence (MDAMKRGLCCVLLLCGAVFVSPSQEIHARFRR) was fused to the N-terminus of all constructs. Mouse-optimized mRNAs with C-terminal oligoDOM™ (GSKKQGDADVCGEVAYIQSVVSDCHVPTAELRTLLEIRKLFLEIQKLKVEGRRRRRS) or an untranslated sequence (STOP-NS: -GSETG-C-RVRRSGIYPERRERLSRSDGRVAHAVGNP-AVLGDSKAQS-GSSSQTF, where each “dash” represents a stop codon) were synthesized by RiboPro B.V. (Oss, The Netherlands) and de-immunized via codon optimization and dsRNA reduction. Each mRNA was equipped with translation-promoting 5' and 3' untranslated regions (UTRs), a 5' anti-reverse capping analogue (ARCA), and a 150A-poly-A-tail. RNA quality was assessed by spectrophotometry and gel electrophoresis. The mRNA was encapsulated in lipid nanoparticles (LNPs) using RiboPro’s ionizable and core lipid in combination with 18:1 (Δ9-Cis) PE (DOPE), 1,2-dioleoyl-3-trimethylammonium propane (DOTAP), and 2-distearoyl-sn-glycero-3-phosphoethanolamine-N-[amino(polyethylene glycol)] (DSPE-PEG), at a charge ratio of (non-polar/polar) of 5. Lipids were dissolved in ethanol and mRNA was dissolved in 100 mM citrate (pH 4.0), and particles were formed by microfluidics at a final concentration of 0.1 µg/µL mRNA. Particles were buffer-exchanged to PBS (pH 7.4) by dialysis. In the last dialysis step, penicillin (100 units/mL)–streptomycin (100 μg/mL) was added to the PBS buffer. All constructs passed RiboPro quality analysis.

**Table 1:** General structure of each construct and epitope sequences used in the study.

Construct ID	Epitope 1	Epitope 2	OligoDOM/STOP-NS
#1-MC38	ELFRAAQLANDVVLQIMEL from Reps1 (CD8) (9)	VHLELASMTNMELMSSIVH from Adpgk (CD8) (9)	OligoDOM
#2-MC38			STOP-NS
#3-CT26	HCWKYLSVQSQLFRGSSLLF from Mitch1 (CD8) (10)	TSKYYMRDVIAIESAWLLEL from Dhx35 (CD4) (10)	OligoDOM
#4-CT26			
			STOP-NS
#5-HPV E7	QAEPDRAHYNIVTFCCKCD from E7 protein (20)	N.A.	OligoDOM
#6-HPV E7			
			STOP-NS
#7-OVA	LEQLESIINFEKLTEWTS from ovalbulmin (CD8) (22)	AESLKISQAVHAAHAEINEAGREVVGS from Ovalbulmin (CD4) (23)	OligoDOM
#8-OVA			
			STOP-NS
#9-unrelated for TC1	RGVQIASNENMETMESSTL from Flu NP (CD8)	N.A.	OligoDOM
#10-unrelated for OVA	RGVQIASNENMETMESSTL from Flu NP (CD8)	LLQNSQVYSLIRPNENPAHKSQLVW from Flu NP (CD4)	OligoDOM

NA, non applicable.

### Mice

Six-week-old female C57BL/6 mice (Charles River Laboratories) were utilized for all experiments, except for testing epitopes from the CT26 colon cancer, where 6-week-old female BALB/c mice (Charles River Laboratories) were used. Animals were kept at the animal houses of OSIVAX (naïve mice experiments), of Turin University (for the experiments with TC-1 cells), and of the University of Grenoble (for the experiments with OVA-expressing B16 tumor). After 7 days of acclimatation, mice were randomly placed in the cages and were housed under specific pathogen-free conditions with ad libitum access to food and water. Ethical approval for animal procedures was obtained from the Institutional Animal Care and Use Committee of the Plateau de Biologie Expérimental de la Souris (CECCAPP_ENS_2022_002, Lyon, France) or DAP # 2018010411002729 of the animal facility at the Faculté de Médecine de Grenoble (France), and the Italian Ministry of Health under reference CC652.124, Aut n° 107/2020. Accreditation was obtained from government agencies.

### Immunizations in naïve mice

The immunogenic response of the mRNA constructs was tested in naïve mice. Five mice per group (80 mice in total) received two immunizations and administered intramuscularly (IM) 3 weeks apart, and PBS was used as negative control. Two doses were used for the OVA constructs (3 and 0.7 µg) as well as for the CT26 constructs (2 and 0.5 µg). One dose at 2 µg was used for the HPV-E7 and MC38 constructs. The immune response was evaluated in the spleens, 7 days after the last immunization, via ELISpot.

### Tumor growth experiments

Two tumor models were selected. For the prophylactic approach, the mouse lung cancer TC-1 cell line (a well-established cancer cell line derived from lung epithelial cells transformed with oncogenic HPV E6 and E7 genes) was used. TC-1 cells (19) were kindly gifted by Prof. Aldo Venuti, Regina Elena National Cancer Institute, Rome. Cells were cultured in RPMI 1640 supplemented with 10% FBS and 400 µg/mL of geneticin (Sigma-Aldrich, Italy) (not included in the last passage before inoculation). Five groups of mice with six mice per group were used (30 mice in total). 2 µg of construct #5 and #6 was administered in groups 1 and 2, respectively, and 0.5 µg of the same constructs #5 and #6 was administered in groups 3 and 4, respectively. An unrelated mRNA construct, i.e., a CD8 epitope from influenza nucleoprotein (Flu NP) fused to oligoDOM™ (construct #9), was used as a negative control and was administered in group 5. All groups were injected IM, 21 days apart. Seven days after the second immunization, 10^5^ TC-1 cells were grafted subcutaneously (SC), and tumor growth was monitored for 25 days. For the therapeutic approach, six groups with six mice per group were used (36 mice in total). 2 × 10^5^ mouse OVA-expressing B16 melanoma cells (B16-OVA) (ATCC^®^ CRL-6475™) were implanted SC, as previously described (20) in all groups. Starting the day after the graft, five immunizations were performed IM, 2 or 3 days apart, with two doses of constructs #7 and #8; group 1 and 2 received 0.7 µg of constructs #7 and #8 respectively, and groups 3 and 4 received 0.2 µg of the same constructs #7 and #8, respectively. An unrelated mRNA construct (CD8 and CD4 Flu NP epitopes fused to oligoDOM™, construct #10 in **Table 1**) was used as negative control and administered to group 6, and 500 µg recombinant OVA (Sigma-Aldrich) formulated with 50 µg of poly IC was used as positive control and administered to group 5. Tumor growth was monitored for 13 days after the graft. Tumor growth was measured as tumor volume, calculated with the following formula: Volume = 0.5 × (Width × Length²). For both experiments, after sacrifice, the spleens were harvested and the immune response was evaluated via ELISpot.

For all experiments, to avoid sample confounders, vaccines were prepared just before the vaccination. To eliminate operator-related confounders, a single operator performed all treatments and measurements. To reduce location-related confounders, all cages were placed at the same level on the rack in a controlled environment (i.e., ventilated, SPF housing), with food, water, and bedding replaced on the same schedule for all cages. The order of treatments and measurements was determined by the initial cage numbering, and no randomization was applied. The tumor size is always measured by the same operator, using an electronic caliper, always measuring the longest side and then the perpendicular (smaller side) in the same way. Experiments were not performed in a blinded manner.

### IFNγ ELISpot

Specific IFNγ-producing splenocytes were enumerated using an IFNγ ELISpot assay (3321-4HPT-10, Mabtech, Sweden). Lymphocytes were isolated from the spleen of individual mice following previously established protocols (12). IFNγ-precoated plates were utilized according to the manufacturer’s instructions (3321-4HPT-10, Mabtech, Sweden). 2.5 × 10^5^ splenocytes were restimulated with the MC38, CT26, E7, or OVA peptides (GenScript, Netherlands) at 2 µg/mL final concentration for 20 h at 37°C/5% CO_2_. Concanavalin A (Sigma-Aldrich, France) served as a positive control, whereas unstimulated splenocytes were used as negative controls. Spot-forming cells (SFCs) were quantified using an ELISpot reader system (ASTOR, Mabtech, Sweden), after background removal.

### Statistical analyses

All statistical analyses and graphical representations were performed using Prism 9.5.1 (GraphPad Software Inc., San Diego, CA). Group comparisons were assessed using an unpaired one-way ANOVA, followed by a Kruskal–Wallis test and Dunn’s multiple comparisons test, excluding the negative control groups. Statistical analysis on the tumor sizes was performed on the final day of the experiment.

Spearman correlation analysis was used to evaluate the relationship between the number of IFNγ-secreting CD8+ T cells in the spleen (post-tumor inoculation and vaccination) and tumor size.

Differences were considered significant if the p value was <0.05. In the figures, the stars were as follows: p < 0.05 (*), p < 0.01 (**), and p < 0.001 (***).

## Results

### OligoDOM™ improved the cell-mediated immune response against short strings of neoepitopes

To create the mRNA constructs where small epitope strings could be fused with oligoDOM™, model epitopes from the literature were selected. Specifically, eight different constructs containing epitopes from four different tumor models were created (**Table 1**), formulated in LNPs, and quality assessed by the vendor (not shown). Two CD8 epitopes were selected from the murine colon carcinoma MC38 (9); two epitopes (CD4 and CD8) from the colon carcinoma CT26 (10) and one CD8 epitope were selected from the E7 protein from HPV (21), a well-known tumor-associated virus (22). Finally, two epitopes were identified from ovalbumin (OVA) as a model antigen expressed by an engineered B16 melanoma cell line (23, 24). The tPA secretion sequence at the N-terminus of the resulting protein was added since preliminary tests in our laboratory showed that a better immune response is triggered by mRNA constructs containing such sequence, compared with similar constructs without any signal sequences (data not shown). Each epitope string was fused at the C-terminus to oligoDOM™ or to a non-translated sequence of the same length (STOP-NS) in the control constructs.

The oligoDOM™-containing mRNA constructs (**Table 1**) were tested in independent experiments in naïve mice. To investigate the immune response, five naïve mice per group were immunized with either one (for MC38 constructs #1 and #2, and HPV-E7 constructs #5 and #6) or two doses of each construct (for the other constructs). The difference in immunization approaches reflects the iterative nature of our experimental design. The CT26 (constructs #3 and #4) and OVA (constructs #7 and #8) experiments were the initial studies performed, during which we aimed to evaluate dose response and determine the better immunization strategy for assessing the constructs’ biological activity. Regarding the dose selection, the initial experiment was conducted with the OVA epitope-containing constructs using two doses (3 µg and 0.7 µg, in a 1:4 ratio). After observing a robust CD8 response and a too low CD4 response at the highest dose, we decided to adjust the doses for the subsequent CT26 study. To optimize the results while maintaining consistency, we reduced the doses but kept the same ratio (1:4). These experiments helped us take the decision to use a higher dose level as overall more relevant for studying the constructs’ biological activity. Therefore, a single dose (2 µg) for the MC38 (#1 and #2) and HPV-E7 (#5 and #6) constructs was deemed sufficient to compare the constructs. This adjustment was made both to ensure that we focused on the most biologically relevant dose and to adhere to the 3Rs principle in animal experimentation, minimizing the number of animals used. The induced cellular immune response was measured via ELISpot and compared with the one induced by similar constructs where the oligoDOM™ sequence was replaced by the STOP-NS sequence (**Figure 1**) to exclude any contribution to the immune response of a simple increase in mRNA content in the oligoDOM™-containing constructs.

Four out of seven oligoDOM™-containing constructs outperformed their counterpart with the STOP-NS sequence (**Figures 1B–E**), achieving up to a 50-fold increase in the immune response for the E7 epitope (**Figure 1D**). Interestingly, oligoDOM™ increased the immune response both for already immunogenic epitopes (**Figures 1B, C, E**) and for those that barely showed immunogenicity in the oligoDOM™-free mRNA construct (**Figure 1D**).

Surprisingly, the CD4 OVA epitope in construct #7 (**Table 1**) showed no dose-dependent effect and no immune response at the highest dose (**Figure 1E**). A similar dose-independent effect was observed for the CD4 epitope from CT26 experiment, with no IFNγ increased response observed at the highest dose (**Figure 1C**).

### OligoDOM™ enhanced the antitumor effect of short strings of neoepitopes in both prophylactic and therapeutic setups

Encouraged by oligoDOM™’s performance in naïve mice, two mouse tumor models were selected to investigate its impact on tumor growth reduction. The TC-1 cancer cell line, derived from lung epithelial cells transformed with oncogenic HPV E6 and E7 genes, and the B16 melanoma cell line expressing OVA were selected for prophylactic and therapeutic treatments, respectively.

**Figure 2:**
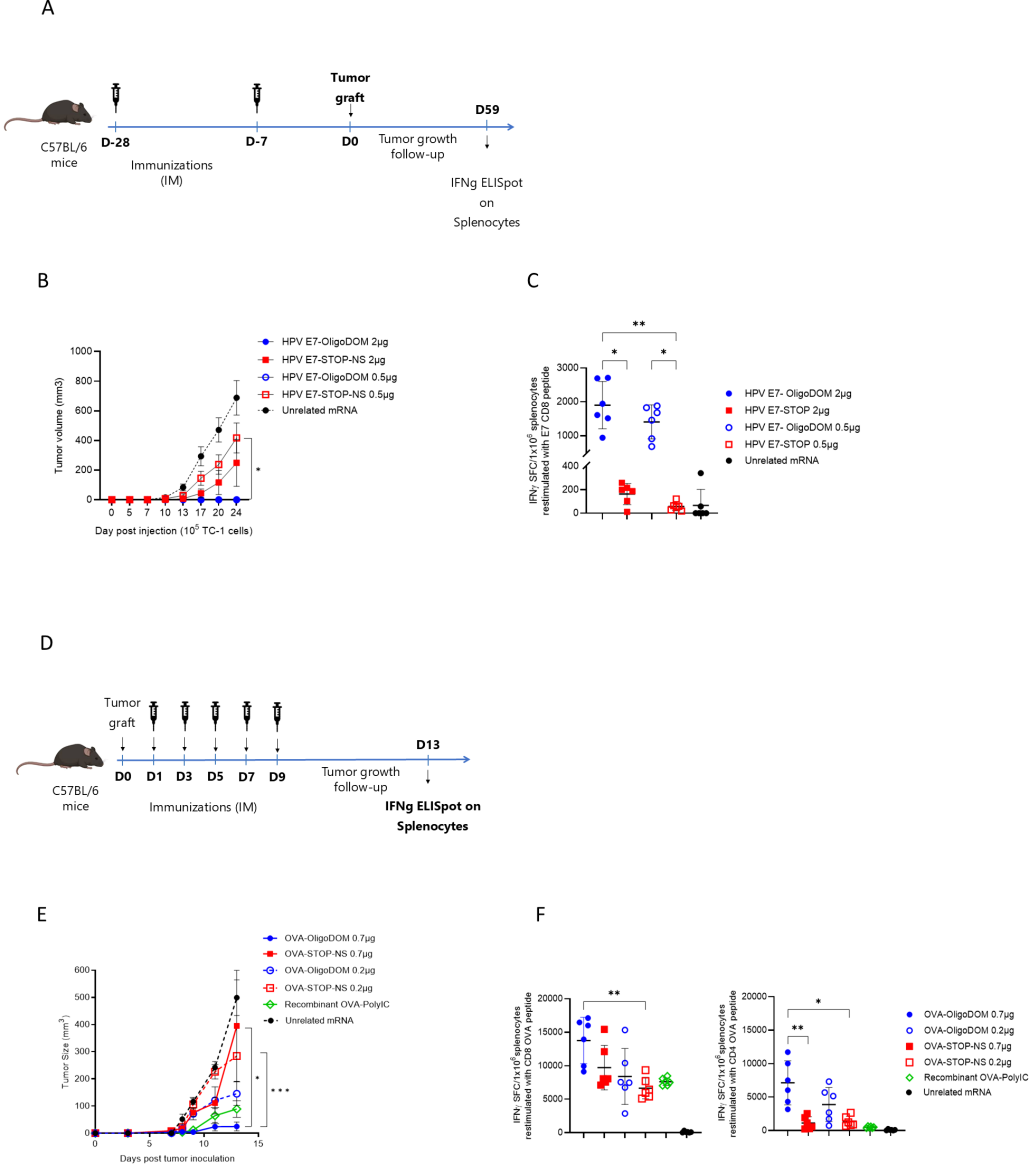
OligoDOM™ in mRNA constructs induces efficient tumor therapy. **(A)** Schematic representation of the prophylactic approach. **(B)** TC-1 tumor growth rate in mice immunized against E7 peptide. Each line refers to the mean tumor volume ± SD of each experimental group (n = 5). **(C)** Immune response measured at the end of the experiment, against the specific E7 epitope used to immunize the mice. E7 specific IFNγ-secreting CD8 T cells were measured by ELISpot (spot-forming cells (SFCs)/1 × 10^6^ cells) in the spleen. Individual data, mean (line), and SD are represented. Differences were assessed by one-way ANOVA followed by Dunn’s multiple comparison test. *p < 0.05; ** p < 0.01. **(D)** Schematic representation of the therapeutic approach. **(E)** B16-OVA tumor growth rate in mice immunized against OVA peptides. Each line refers to the mean tumor volume ± SD of each experimental group (n = 5). **(F)** Immune response against OVA epitopes at the end of the experiment. CD8- or CD4- peptide specific IFNγ secreting T-cells (spot-forming cells (SFCs)/1 × 10^6^ cells) in the spleen. Individual data, mean (line), and SD were represented. Differences were assessed by one-way ANOVA followed by Dunn’s multiple comparison test. *p < 0.05; ** p < 0.01.

The mRNA constructs used for these experiments were HPV-E7 #5 and OVA #7 (**Table 1**), their STOP-NS counterparts (constructs #6 and #8), and the unrelated Flu NP epitope string fused with oligoDOM™ (negative control, #9 and #10). The HPV-E7-CD8-epitope mRNA constructs (#5 and #6) were administered with the same immunization schedule as in the previous experiment with naïve mice (**Figure 2A**). This time, two doses (2 and 0.5 µg) were used to test a potential dose-dependent effect. TC-1 cells were grafted SC 7 days after the immunization cycle, and tumor growth was monitored for 25 days. Complete tumor growth prevention was observed in mice immunized with the oligoDOM™-containing construct at both doses, whereas only partial growth reduction was observed in mice immunized with the HPV-E7 construct #6 (without oligoDOM™) at both doses when compared with the negative control (sequence #9) (**Figure 2B**). The statistical analysis revealed a significant difference between the groups immunized with both doses of the HPV-E7-oligoDOM™ construct (construct #5) and the group receiving the construct without oligoDOM™ (construct #6) at 0.5 µg (*p* < 0.05, *) (**Figure 2B**). The tumor rejection observed (at both doses tested) was paralleled by the induction of a T-cell immune response against the E7 peptide approximately 10 times stronger with the oligoDOM™-containing constructs than with the mRNA sequence without oligoDOM™ (**Figure 2C**), measured via ELISpot assay in the spleens of these mice at the end of the experiment.

For the therapeutic setup experiment, the mouse melanoma B16-OVA model was used to compare the antitumor efficacy of the OVA constructs #7 and #8 (**Table 1**). B16-OVA SC cells were implanted into mice, and starting the day after engraftment, the animals received five immunizations (**Figure 2D**). In this experiment, the most pronounced reduction in tumor growth rate was observed in mice immunized with the OVA-oligoDOM™ construct. Notably, four out of six mice in the highest-dose group (OVA construct #7) remained tumor-free, whereas only two out of six mice in the positive control group, which received recombinant OVA with a Poly-IC adjuvant, exhibited a tumor-free status (**Figure 2E**). In contrast, no significant reduction in tumor growth rate was detected in the negative control group administered construct #10 (**Figure 2E**). Statistical analysis demonstrated a significant difference in tumor growth rates between the highest dose of the OVA-oligoDOM™ construct (#7) at 0.7 µg and both doses of the OVA construct lacking oligoDOM. Restimulated splenocytes from mice immunized with the oligoDOM™-containing mRNA constructs showed a higher number of IFNγ releasing specific T cells. Interestingly, in the presence of the OVA-expressing tumor, also the CD4 signal was detected and superior in the oligoDOM™-treated group, unlike what was observed with the naïve mice (**Figures 1E**, **2F**).

**Figure 3:**
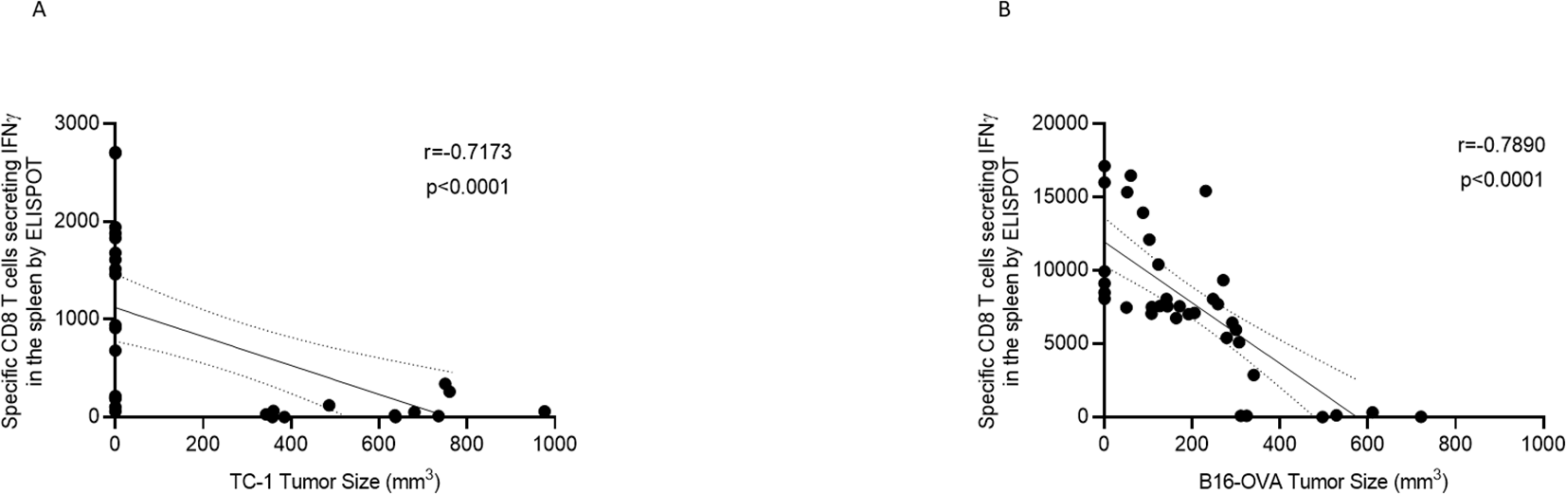
A strong link between the increased immune response triggered by oligoDOM™ and the efficacy against tumor growth is evident. The Spearman correlation graphs illustrate the relationship between specific T cells secreting IFNγ in the spleen and the tumor size across all groups in the HPV prophylactic experiment **(A)** and OVA therapeutic experiment **(B)**.

A Spearman correlation test was performed to determine the relationship between the T-cell response triggered by vaccination and tumor size at the end of the experiments for both the prophylactic and the therapeutic setups. In both cases, we observed a correlation between the specific immune response measured with ELISpot and the size of the tumors at sacrifice, with a correlation coefficient of −0.7173 for the prophylactic experiment and −0.7890 for the therapeutic approach. The higher the immune response, the smaller the tumor size at the end of the experiment (**Figure 3**).

Overall, the antitumor effect of the oligoDOM™-containing constructs was higher for both tumor models, in both prophylactic and therapeutic setups.

## Discussion

In this work, we aimed to demonstrate the added value of the self-assembling oligoDOM™ sequence in triggering a specific and effective immune response when fused to tumor neoantigen strings in an mRNA construct formulated in LNPs.

Using short epitope strings of one or two epitopes as model sequences, either alone or fused to oligoDOM™, we successfully showed the benefits of oligoDOM™ in both naïve mice, focusing on the specific cell-mediated immune response triggered, and in tumor-bearing mice, focusing on the reduction of tumor growth. Indeed, in both experimental setups, an increased immune response and an antitumor efficacy were observed in the mice treated with the oligoDOM™-containing constructs, compared with those immunized with constructs without oligoDOM™.

The correlation between the intensity of the specific immune response (measured as IFNγ-secreting splenocytes) and the effect on tumor growth, in both therapeutic and prophylactic setups, confirmed a direct effect of the elicited specific T cells against the neoepitopes used in the constructs. Interestingly, not only did oligoDOM™ increase the immune response against already immunogenic neoepitopes but also it converted one poorly immunogenic epitope into highly immunogenic one (e.g., the HPV-E7 epitope used in this study, 20). This led to the generation of specific T cells capable of slowing down or even abolishing the growth of tumors bearing that specific neoepitope.

The effect of oligoDOM™ fused to the neoepitopes was more pronounced for the tested CD8 epitopes and less so for CD4 epitopes in naïve mice. This is a known phenomenon described in the literature, where higher antigen doses can suppress CD4 activation while still showing dose-dependent CD8 activation in an antigen-dependent manner (25). However, in the B16-OVA tumor model, we demonstrated that when the B16-OVA tumor was grafted, the immune response against the OVA CD4 epitope was also increased when mice were immunized with constructs fused with oligoDOM™, contributing to the overall reduction in tumor growth rate.

To test the performance of the oligoDOM™ platform to control tumor growth, a prophylactic approach and a therapeutic approach were put in place. For the prophylactic approach, we used the virus-induced TC-1 tumor model expressing the HPV-E7 protein. This model allowed us to evaluate the platform’s ability to elicit a strong preventive immune response against a defined, exogenous antigen in a well-characterized system. In contrast, the therapeutic approach was tested using the B16 melanoma model, widely recognized for its relevance to immunotherapy research due to its aggressive nature and immunosuppressive environment. Despite the limitation on directly comparing the two strategies, using two different tumor models enabled us to address different aspects of the platform’s efficacy (prophylactic and therapeutic). OligoDOM™ conferred effective tumor growth reduction in both tumor models investigated when mice were treated with short epitope strings. This is notable, as such short epitope strings often result in poor effectiveness in the presence of tumors in preclinical models when administered as nucleic acids (9).

Our goal here was not to set specific dose thresholds or a specific combination or number of neoantigens, but to demonstrate the superiority, by direct comparison, of mRNA-based vaccines bearing the oligoDOM™ sequence over the same constructs lacking this sequence. The TC-1 and OVA-B16 tumor models were chosen as a pilot approach to study the underlying mechanisms of the oligoDOM™ Platform and to establish a proof of concept for its ability to enhance T-cell responses against small epitope strings and correlate it with tumor growth reduction. Specifically, we aimed to demonstrate that constructs containing one or two small neoepitopes could significantly slow tumor growth, assuming their presence in the tumor. Considering the very high immune response generated and the efficacy data recorded in the mouse models and experimental setups investigated in this work, our findings support the idea that oligoDOM™ could be of great utility in cases in which short epitope strings may need to be used for personalized therapy, and where tumor-associated mutations may show poor immunogenicity. Our conclusions are based on proof-of-concept findings in artificial models: further studies are required to validate the platform’s effectiveness in more relevant systems, particularly with true tumor-specific epitopes of low mutational burden tumors.

The genetic analysis of a tumor can lead to the identification of specific tumor-associated mutations, and the sequences bearing such mutations can be included in an mRNA formulation as part of an immune therapy (6–8). Fusing the oligoDOM™ sequence to a string of epitopes in an mRNA construct is relatively fast from a technical point of view. Our findings, once confirmed, could pave the way to a clinical scenario in which the identification of tumor-associated mutations for therapeutic purposes could be coupled to the oligoDOM™ technology to obtain tumor targeted therapeutic programs, where an enhanced targeted immune response could be key for the success of the treatment.

## Data Availability

The raw data supporting the conclusions of this article will be made available by the authors, without undue reservation.
