# Supplementary material for: OligoDOMTM: a T-cell response-enhancing platform applied to cancer immunotherapy
Source: Front Immunol. 2025 Mar 14;16:1549112. doi: 10.3389/fimmu.2025.1549112 (PMC11951937; doi:10.3389/fimmu.2025.1549112)
Supplement: Supplementary file 1 [file Table1.pdf]

Supplementary Material

Supplementary Table 1: mRNA sequences of the encoding portion of each constructs used in this work.

**Construct ID**	**Full mRNA sequence of the encoding portion of each construct**
#1-MC38	AUGGACGCCAUGAAGAGGGGCCUGUGCUGCGUGCUGCUGCUGUGCGGC GCCGUGUUCGUGAGCCCUAGCCAGGAGAUCCACGCCAGGUUCAGGAGG GAGCUGUUCAGGGCCGCCCAGCUGGCCAACGACGUGGUGCUGCAGAUC AUGGAGCUGGUGCACCUGGAGCUGGCCAGCAUGACCAACAUGGAGCUG AUGAGCAGCAUCGUGCACGGCAGCAAGAAGCAGGGCGACGCCGACGUG UGCGGCGAGGUGGCCUACAUCCAGAGCGUGGUGAGCGACUGCCACGUG CCUACCGCCGAGCUGAGGACCCUGCUGGAGAUCAGGAAGCUGUUCCUG GAGAUCCAGAAGCUGAAGGUGGAGGGCAGGAGGAGGAGGAGGAGCUGA
#2-MC38	AUGGACGCCAUGAAGAGGGGCCUGUGCUGCGUGCUGCUGCUGUGCGGC GCCGUGUUCGUGAGCCCUAGCCAGGAGAUCCACGCCAGGUUCAGGAGG GAGCUGUUCAGGGCCGCCCAGCUGGCCAACGACGUGGUGCUGCAGAUC AUGGAGCUGGUGCACCUGGAGCUGGCCAGCAUGACCAACAUGGAGCUG AUGAGCAGCAUCGUGCACUGAGGCAGCGAGACCGGCUGAUGCUGAAGG GUGAGGAGGAGCGGCAUCUACCCUGAGAGGAGGGAGAGGCUGAGCAGG AGCGACGGCAGGGUGGCCCACGCCGUGGGCAACCCUUGAGCCGUGCUG GGCGACAGCAAGGCCCAGAGCUGAGGCAGCAGCAGCCAGACCUUCUGA
#3-CT26	AUGGACGCCAUGAAGAGGGGCCUGUGCUGCGUGCUGCUGCUGUGCGGC GCCGUGUUCGUGAGCCCUAGCCAGGAGAUCCACGCCAGGUUCAGGAGG ACCAGCAAGUACUACAUGAGGGACGUGAUCGCCAUCGAGAGCGCCUGG CUGCUGGAGCUGCACUGCUGGAAGUACCUGAGCGUGCAGAGCCAGCUG UUCAGGGGCAGCAGCCUGCUGUUCGGCAGCAAGAAGCAGGGCGACGCC GACGUGUGCGGCGAGGUGGCCUACAUCCAGAGCGUGGUGAGCGACUGC CACGUGCCUACCGCCGAGCUGAGGACCCUGCUGGAGAUCAGGAAGCUG UUCCUGGAGAUCCAGAAGCUGAAGGUGGAGGGCAGGAGGAGGAGGAGG AGCUGA
#4-CT26	AUGGACGCCAUGAAGAGGGGCCUGUGCUGCGUGCUGCUGCUGUGCGGC GCCGUGUUCGUGAGCCCUAGCCAGGAGAUCCACGCCAGGUUCAGGAGG ACCAGCAAGUACUACAUGAGGGACGUGAUCGCCAUCGAGAGCGCCUGG CUGCUGGAGCUGCACUGCUGGAAGUACCUGAGCGUGCAGAGCCAGCUG UUCAGGGGCAGCAGCCUGCUGUUCUGAGGCAGCGAGACCGGCUGAUGC UGAAGGGUGAGGAGGAGCGGCAUCUACCCUGAGAGGAGGGAGAGGCUG AGCAGGAGCGACGGCAGGGUGGCCCACGCCGUGGGCAACCCUUGAGCC GUGCUGGGCGACAGCAAGGCCCAGAGCUGAGGCAGCAGCAGCCAGACC UUCUGA

#5-HPV E7	AUGGACGCCAUGAAGAGGGGCCUGUGCUGCGUGCUGCUGCUGUGCGGC GCCGUGUUCGUGAGCCCUAGCCAGGAGAUCCACGCCAGGUUCAGGAGG CAGGCCGAGCCUGACAGGGCCCACUACAACAUCGUGACCUUCUGCUGCA AGUGCGACGGCAGCAAGAAGCAGGGCGACGCCGACGUGUGCGGCGAGG UGGCCUACAUCCAGAGCGUGGUGAGCGACUGCCACGUGCCUACCGCCGA GCUGAGGACCCUGCUGGAGAUCAGGAAGCUGUUCCUGGAGAUCCAGAA GCUGAAGGUGGAGGGCAGGAGGAGGAGGAGCUGA
#6-HPV E7	AUGGACGCCAUGAAGAGGGGCCUGUGCUGCGUGCUGCUGCUGUGCGGC GCCGUGUUCGUGAGCCCUAGCCAGGAGAUCCACGCCAGGUUCAGGAGG CAGGCCGAGCCUGACAGGGCCCACUACAACAUCGUGACCUUCUGCUGCA AGUGCGACUGAGGCAGCGAGACCGGCUGAUGCUGAAGGGUGAGGAGGA GCGGCAUCUACCCUGAGAGGAGGGAGAGGCUGAGCAGGAGCGACGGCA GGGUGGCCCACGCCGUGGGCAACCCUUGAGCCGUGCUGGGCGACAGCA AGGCCCAGAGCUGAGGCAGCAGCAGCCAGACCUUCUGA
#7-OVA	AUGGACGCCAUGAAGAGGGGCCUGUGCUGCGUGCUGCUGCUGUGCGGC GCCGUGUUCGUGAGCCCUAGCCAGGAGAUCCACGCCAGGUUCAGGAGG CUGGAGCAGCUGGAGAGCAUCAUCAACUUCGAGAAGCUGACCGAGUGG ACCAGCGCCGAGAGCCUGAAGAUCAGCCAGGCCGUGCACGCCGCCCACG CCGAGAUCAACGAGGCCGGCAGGGAGGUGGUGGGCAGCGGCAGCAAGA AGCAGGGCGACGCCGACGUGUGCGGCGAGGUGGCCUACAUCCAGAGCG UGGUGAGCGACUGCCACGUGCCUACCGCCGAGCUGAGGACCCUGCUGG AGAUCAGGAAGCUGUUCCUGGAGAUCCAGAAGCUGAAGGUGGAGGGCA GGAGGAGGAGGAGGAGCUGA
#8-OVA	AUGGACGCCAUGAAGAGGGGCCUGUGCUGCGUGCUGCUGCUGUGCGGC GCCGUGUUCGUGAGCCCUAGCCAGGAGAUCCACGCCAGGUUCAGGAGG CUGGAGCAGCUGGAGAGCAUCAUCAACUUCGAGAAGCUGACCGAGUGG ACCAGCGCCGAGAGCCUGAAGAUCAGCCAGGCCGUGCACGCCGCCCACG CCGAGAUCAACGAGGCCGGCAGGGAGGUGGUGGGCAGCUGAGGCAGCG AGACCGGCUGAUGCUGAAGGGUGAGGAGGAGCGGCAUCUACCCUGAGA GGAGGGAGAGGCUGAGCAGGAGCGACGGCAGGGUGGCCCACGCCGUGG GCAACCCUUGAGCCGUGCUGGGCGACAGCAAGGCCCAGAGCUGAGGCA GCAGCAGCCAGACCUUCUGA
#9-unrelated for TC1	AUGCGUGGUGUGCAAAUCGCCAGCAACGAAAACAUGGAAACCAUGGAA UCUUCAACCCUGGGCAGCAAGAAACAGGGUGAUGCUGACGUGUGCGGC GAAGUGGCAUAUAUCCAGAGCGUCGUGAGCGAUUGUCACGUUCCGACG GCAGAGUUGCGCACGCUGUUGGAAAUCCGUAAGCUGUUCUUGGAGAUU CAAAAGCUCAAAGUUGAGGGUCGUCGUCGCAGACGUUCCUAA
#10-unrelated for OVA	AUGGAUGCAAUGAAGAGAGGGCUCUGCUGUGUGCUGCUGCUGUGUGGA GCAGUCUUCGUUUCGCCCAGCCAGGAAAUCCAUGCCCGAUUCAGAAGA CGUGGUGUGCAAAUCGCCAGCAACGAAAACAUGGAAACCAUGGAAUCU

	UCAACCCUGCUGCUGCAGAACAGCCAGGUGUACAGCCUGAUCCGUCCAA<br>AUGAGAACCCGGCACACAAGUCCCAACUGGUUUGGGGCAGCAAGAAAC<br>AGGGUGAUGCUGACGUGUGCGGCGAAGUGGCAUAUAUCCAGAGCGUCG<br>UGAGCGAUUGUCACGUUCCGACGGCAGAGUUGCGCACGCUGUUGGAAA<br>UCCGUAAGCUGUUCUUGGAGAUUCAAAAGCUCAAAGUUGAGGGUCGUC<br>GUCGCAGACGUUCCUAA
